# Physical exercise as a precision strategy for targeted PINK1 recruitment- a mechanistic review

**DOI:** 10.3389/fphys.2026.1836651

**Published:** 2026-06-08

**Authors:** Jing Zhu, Ligang Tong, Anand Thirupathi

**Affiliations:** 1 Ningbo Childhood Education College, Ningbo, China; 2 Faculty of Sports Science, Ningbo University, Ningbo, China

**Keywords:** mitochondria, mitophagy, neurons, Parkin, physical exercise, PINK1

## Abstract

PTEN-induced kinase 1 (PINK1) is a mitochondrial serine/threonine kinase that orchestrates ubiquitin-dependent mitophagy together with the E3 ligase Parkin. Both physiological and pathological conditions rapidly recruit PINK1, and timely PINK1 degradation in healthy mitochondria determines whether it supports or harms the cell. Thus, the tight regulation of PINK1 balances its negative effects. In this context, introducing physical exercise as one of the strategies can fine-tune PINK1/Parkin pathways by triggering transient energy stress and moderate increases in reactive oxygen species (ROS) that promote PINK1 stabilization on the outer mitochondrial membrane, enhance Parkin recruitment via sensitizing various molecular signaling, such as AMPK–PGC-1α and FOXOs. However, the mechanism underlying a specific exercise mode that triggers PINK1-mediated selective removal of mitochondrial damage remains unknown. Therefore, this review will synthesize mechanistic approaches to how different exercise paradigms modulate PINK1 function, recruit PINK1 dynamics, and regulate downstream signaling, to define exercise prescriptions as adjunctive strategies.

## 1 Introduction

Although the fundamental concept of all the molecular proteins has been well studied for over a century, the discovery of these protein interactions continues to reveal the limitations of traditional molecular biology. PTEN-induced kinase 1 (PINK1) is a recent prime example, initially identified for its role in ovarian cancer (Unoki and Nakamura, 2001). Shortly afterward, this protein was recognized as a crucial factor in the early onset of Parkinson’s disease (PD) (Valente et al., 2004), indicating that specific molecular proteins, like PINK1, shattered the conventional view that these proteins act as linear signaling pathways, often as direct signaling pathways. A recent study also confirmed this node that the discovery of PINK1 interactions not only with Parkin but also with dynamin-related protein 1 (Drp1) and protein kinase A (PKA) to regulate mitochondrial dynamics, revealing PINK1 as a central node within the jumble of molecular signaling web, and each newly identified PINK1 target highlights the multitasking role of PINK1 across various signaling pathways (Das Banerjee et al., 2017; Gao et al., 2022). Additionally, this can make it difficult to predict PINK1’s role in overall cellular functions using current static models. Most importantly, these models failed to capture real-time cellular signaling of PINK1, such as “Where” and “When”. For instance, cleaved PINK1 fragments in chronic metabolic diseases can cross into the nucleus. This scenario can cause unintended gene expression before they are eliminated (Lazarou et al., 2012), highlighting the need for a specific external factor, such as physical exercise, that can strictly regulate their damage-sensing abilities. Furthermore, real-time capture of PINK1 transient signaling with large multimeric structures, such as the translocase of the Outer Membrane-voltage-dependent anion channel (TOM-VDAC) complex, remains challenging.

To address these limitations, physical exercise can offer a real-time physiological model for understanding transient molecular events. Primarily, exercise research has fundamentally advanced our understanding of how biochemical pathways and subsequent molecular protein interactions are activated in response to the physiological status of the individual. This paradigm was initiated by the work of John O. Holloszy, who was a pioneer in identifying how biochemical adaptation occurs during exercise in skeletal muscle and subsequently established that exercise acts as a precise molecular signal activator, effectively establishing the field of molecular exercise physiology (Holloszy and Narahara, 1965). Since then, several studies have detailed how exercise activates or suppresses different molecular proteins in different conditions. In the context of transient PINK1 activation, acute exercise acts as a physiological “reset button, ” altering mitochondrial membrane potential and modulating TOM complex assembly (Takahashi and Hood, 2012; Callegari et al., 2025). Thus, it drives the formation of the PINK/TOM/VDAC complex, which rapidly recruits PINK1 and stabilizes its kinase domain outward toward the cytosol. This scenario offers a crucial “Landing Pad” for Parkin. Beyond this, exercise has been reported to influence multiple cellular redox processes that directly impact PINK1 activation (Liu et al., 2025; Ji et al., 2020), highlighting the therapeutic connection between exercise-induced redox signaling and the PINK1 pathway in human diseases.

Despite these exercise-mediated advantages for PINK1 regulation, the specific mechanism by which PINK1 drives mitophagy remains unclear. Furthermore, many excellent reviews have comprehensively detailed exercise-mediated general redox biology, skeletal muscle adaptation, and intracellular signaling (Ji et al., 2020; Powers and Jackson, 2008). Nevertheless, the specific molecular interactions of PINK1 across different exercise modes remain underexplored. Therefore, this review examines how exercise affects the functional components of PINK1 by modulating redox events-mediated transient molecular interactions. We primarily aim to address the underlying mechanisms of exercise and PINK1, synthesize structural and physiological evidence, and propose new research hypotheses and outline future research directions for targeting PINK1 in metabolic and neurodegenerative diseases.

## 2 General aspects of PINK1 dynamics and regulation

Under physiological conditions, PINK1 is tightly regulated at low levels through continuous import, cleavage, and cytosolic degradation. Full-length PINK1 is initially imported through the TOM complex. Once PINK1`s N-terminal sequence reaches the inner mitochondrial membrane, it can undergo PARL-mediated cleavage, and the resulting fragments act as N-degrons. Following this, truncated PINK1 is retro-translocated to the cytosol, where it rapidly undergoes proteasomal degradation via the N-end rule pathway (Jin et al., 2010). Conversely, under stress conditions, such as intense physical exercise-induced transient energy stress or ROS elevation, mitochondrial membrane potential is disrupted. When mitochondria are damaged, PINK1 can no longer be translocated from the outer membrane to the inner mitochondrial membrane (Quinn et al., 2020; Sun et al., 2025). Because it fails to reach the PARL cleavage system, resulting in full-length PINK1 stabilization and accumulation on the outer mitochondrial membrane, serving as a critical molecular tag that recruits Parkin to initiate clearance of damaged mitochondria and help maintain cellular homeostasis during post-exercise recovery (Quinn et al., 2020; Sun et al., 2025; Zhao et al., 2023). So, understanding these foundational dynamics could provide a conceptual framework for establishing how specific exercise protocols can strategically support the PARL cleavage system to drive targeted, adaptive mitophagy.

## 3 Exercise-induced regulation of PINK1 stability and degradation

Concededly, elucidating the molecular regulators of PINK1 stability and degradation during exercise presents significant challenges. This review considers any mechanism disrupting mitochondrial homeostasis through PINK1 as a principal exercise-mediated modulator of PINK1 stability and degradation. The maladaptive accumulation of reactive oxygen species (ROS) and subsequent collapse of the mitochondrial membrane potential (Δψm) are crucial mechanisms that disrupt PINK1 cleavage, leading to aberrant recognition by mitochondrial proteases such as PARL and over-cleavage of PINK1 (Deas et al., 2011). Mechanistically, exercise favors blocking over-cleavage enhancement by reducing maladaptive ROS accumulation (Kolodziej and O'Halloran, 2021), and by inhibiting PINK1 import via PARL blockade, resulting in reducing PINK1 cleavage and stabilizing full-length PINK1, triggering adaptive mitophagy (Díaz-Castro et al., 2024). The N-end rule and endoplasmic reticulum-associated protein degradation (ERAD) pathways may also destabilize PINK1 following its post-cleavage in the mitochondria (Guardia-Laguarta et al., 2019; Yamano and Youle, 2013). For example, exercise-induced acute decrease in methionine levels may affect the N-end rule pathway (Heo et al., 2023), thereby making PINK1 unstable if it is not processed by PARL. In contrast, increased ROS formation leads to methionine oxidation, including methionine sulfoxide formation, which impairs exercise endurance (Lai et al., 2019). Also, exercise-induced ROS, such as hydrogen peroxide (H2O2), oxidize cysteine. This can promote arginylation to form Arg-CysO2(H), which acts as an Arg/N-degron for ubiquitylation and subsequent degradation by ubiquitin-protein ligase E3 component n-recognin 1 and 2 (UBR1 and UBR2) (Liu et al., 2025). For example, Sekine and Youle reported that the Phe-Pink1 Ct-fragment is retrotranslocated from the mitochondria to the cytosol, where the Arg/N-degron degrades PINK1 (Sekine and Youle, 2018). Conceptually, this mechanism could regulate OMM-bound uncleaved Pink1 and maintain mitochondrial homeostasis (Sekine and Youle, 2018; Varshavsky, 2019). However, the effect of exercise on this mechanism requires further investigation. Exercise-induced cysteine oxidation may also promote homo-dimerization, initiating downstream signaling and inducing PINK1 kinase activity (Okatsu et al., 2018; Callegari et al., 2025). For instance, the oxidation of cysteine to lower oxidation states, particularly exercise-induced reductions in H2O2 levels with a rate constant up to 10^8 M−1 s−1 in proteins like PINK1, results in reversible oxidation of cysteine thiol. This can lead to the formation of either inter- or intramolecular disulfides, which are essential for PINK1 stabilization and the initiation of mitophagy in Parkinson’s disease (Wadley et al., 2016; Arena et al., 2021; García-Santamarina et al., 2014). While these degradation pathways govern baseline mitochondrial homeostasis in peripheral tissues, including skeletal muscle, the high-energy demands of the CNS present a specific metabolic environment. Consequently, understanding how these pathways operate in neurons during exercise is critical, as explored in the following section.

## 4 Exercise breaks the PINK1 oxidation vicious cycle in neurons

**Figure 1:**
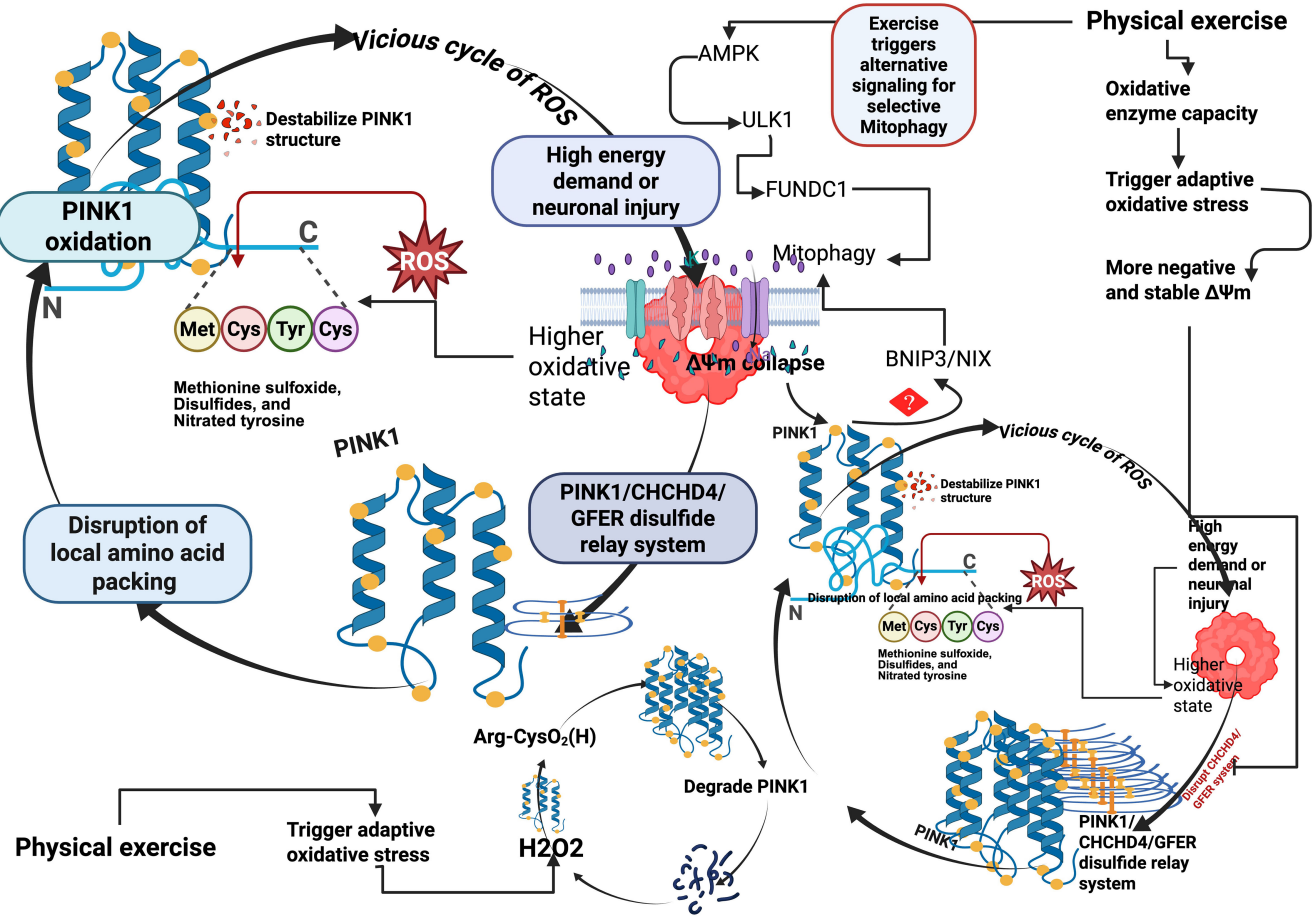
Physical exercise disrupts the vicious oxidation cycle of PINK1. High energy demand collapse ΔΨm, which accelerates the oxidation of amino acids like Met, Cys, and Tyr, causes helix–coil imbalance in the PINK1 structure, leading to its destabilization. Oxidants such as H2O2 promote Arg-CysO2(H), functioning as an Arg/N-degron to regulate PINK1 degradation. Additionally, exercise activates alternative mitophagy pathways involving AMPK, ULK1, and FUNDC1, functioning independently of PINK1. Moreover, adaptive oxidative stress from physical activity inhibits the PINK1/CHCHD4/GFER disulfide relay system, affecting proper PINK1 accumulation.

Sustained high energy demand in neurons reshapes the protein oxidation landscape, including that of PINK1. Amphipathic helix residues in PINK1, such as cysteine, methionine, and tyrosine, are particularly susceptible to oxidants, yielding byproducts like methionine sulfoxide, disulfides, and nitrated tyrosine that destabilize local amino acid packing within the helix and shift the helix–coil equilibrium (**Figure 1**) (Gautier et al., 2008). Following neuronal injury or extreme energy demand, oxidative stress triggers aberrant disulfide bond formation, disrupting the PINK1/CHCHD4/GFER disulfide relay system and impairing PINK1 accumulation and mitophagy (Barodia et al., 2017; Gao et al., 2020). The result is a vicious cycle that exacerbates PINK1 oxidation and drives further pathology.

Although direct evidence in human neuronal models is currently lacking, emerging conceptual frameworks suggest that exercise might interrupt this vicious cycle by triggering controlled adaptive oxidative stress by enhancing the capacity of oxidative enzymes and the electron transport chain (ETC) efficiency, helping to maintain a more negative and stable ΔΨm for fine-tuning PINK1 function, and restoring mitochondrial quality control without overwhelming the intermembrane space (IMS) disulfide relay (Zorova et al., 2018). Additionally, exercise supports other adaptive mechanisms independent of PINK1/Parkin by recruiting alternative mitophagy-related signaling pathways, including AMP-activated protein kinase/Unc-51-like kinase 1 (AMPK/ULK1), BNIP3/NIX, and FUN14 domain-containing protein 1 (FUNDC1) (Laker et al., 2017; Drake et al., 2019), via hypoxia-inducible factor-1α (HIF-1α) and Forkhead box O3 (FOXO3). In particular, when exercise triggers these alternative mitophagy-related signaling pathways independent of PINK1/Parkin (Gao et al., 2025), neurons can efficiently cope with their high energy demands, rather than shifting to glycolysis, to which they are particularly vulnerable, and regular exercise keeps the neurons’ oxidative environment within limits (Markaki et al., 2023; Basak and Holzbaur, 2025). Physical exercise improves mitochondrial efficiency in neurons and reduces their susceptibility to depolarization (Van Laar and Berman, 2013); consequently, basal Parkin translocation can decline simply because fewer damaged mitochondria require tagging. This mechanism inhibits oxidative stress by reducing ROS leakage, enhancing antioxidant buffering capacity, and stabilizing the membrane potential (Steiner et al., 2011).

## 5 How physical exercise regulates PINK1 transcription

**Figure 2:**
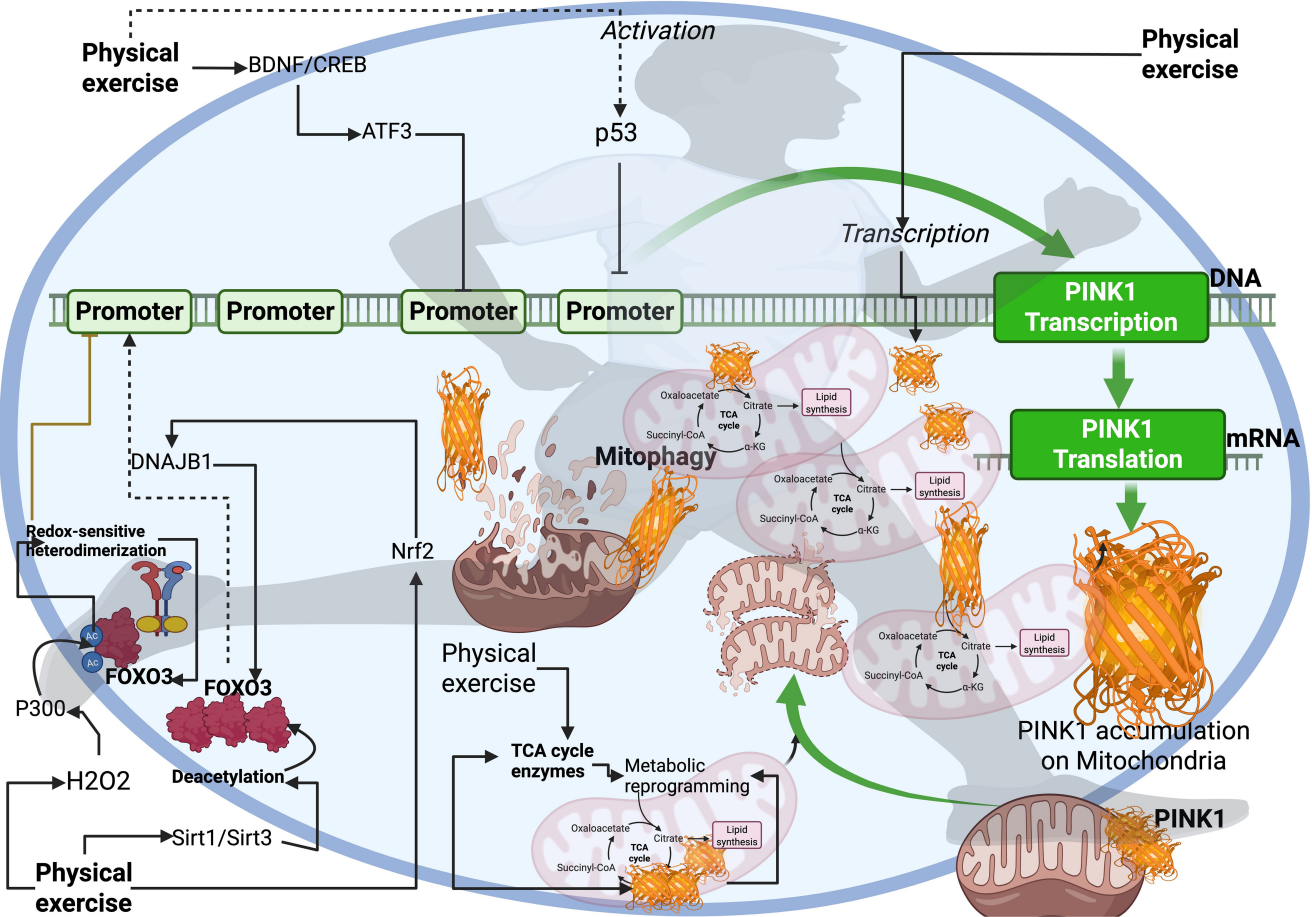
Mechanism of PINK1 transcription and mitochondrial regulation during. Physical exercise-induced deacetylation of FOXO3 by Sirt1/Sirt3 induces transcriptional activity of PINK1. Exercise alters TCA cycle enzymes by modulating PINK1, promoting metabolic reprogramming, and underscoring PINK1’s role beyond mitophagy. Exercise activates BDNF/CREB/ATF3 for PINK1 transcription. Also, exercise-induced Nrf2 targets DNAJB1, activating FOXO3, and promoting PINK1 transcription. Also, exercise-induced redox-sensitive heterodimerization induces PINK1 transcription by H2O2-mediated P300 activation.

Although the transcriptional regulation of PINK1 has been explored, the mechanisms that regulate its post-transcriptional activation remain unclear. Current evidence indicates that physical exercise can influence enzymes and several transcription factors that regulate PINK1 expression (Chen et al., 2018). For instance, voluntary wheel running increases apurinic/apyrimidinic endonuclease 1 (APE1) via Brain-derived neurotrophic factor/cyclic-AMP response element-binding (BDNF/CREB) signaling, thereby promoting PINK1 transcription (Yang et al., 2014). APE1 may also affect nuclear factor kappa B (NF-κB), which binds to multiple cis-acting sites in the PINK1 promoter to upregulate PINK1. This may be due to the early adaptive response to exercise, as NF-κB activity appears to decline with prolonged light-to-moderate exercise (Duan et al., 2014). Nevertheless, the net effect of exercise intensity on PINK1 expression remains to be determined in this context. Physical exercise can further affect PINK1 through redox-sensitive and myogenic transcriptional programs (Tang et al., 2024). For example, accumulation of nuclear factor erythroid 2-related factor 2 (Nrf2) during exercise increases the antioxidant response element (ARE). This can increase PINK1 promoter activity in neuronal cells (**Figure 2**) (Murata et al., 2015; Bento-Pereira and Dinkova-Kostova, 2021). Exercise also induces the DnaJ heat shock protein family member B1 (DNAJB1/hsp40), fibronectin 1, and LIM domain and actin-binding 1 (LIMA1) (Anderson et al., 2021), which can modulate FOXO3, a factor known to promote PINK1 transcription (Mei et al., 2009). For instance, exercise-induced ROS, mainly H_2_O_2_ at concentrations of 25–500 μM, induce acetyltransferase interaction with FOXO3 for its maximum activity (Antunes and Brito, 2017; Essers et al., 2004; Gupta et al., 2022). Although this concentration is physiologically lethal (physiological range: 0.1–7 μM), it can induce adaptive responses to oxidative stress and trigger redox-sensitive heterodimerization via cysteine residues, forming intermolecular disulfide bridges (Antunes and Brito, 2017). This can prevent FOXO3 binding to the PINK1 promoter and reduce PINK1-dependent mitophagy. In contrast, exercise induces deacetylation of FOXO3 by SIRT1 and SIRT3. This can restore FOXO3 binding to the PINK1 promoter and trigger PINK1-mediated mitophagy (Furrer and Handschin, 2024). Exercise can engage PINK1 transcriptional repressors, such as activating transcription factor 3 (ATF3), which has been implicated in ER-stress–mediated repression of PINK1 and disruption of mitochondrial homeostasis (Liu and Chang, 2018; Fernández-Verdejo et al., 2017; Bueno et al., 2018), and exercise can also activate p53, whose accumulation has been shown to suppress PINK1 expression (Goiran et al., 2018; Marchenko and Moll, 2014).

## 6 Does exercise type affect mitochondrial quality via activating PINK1?

**Figure 3:**
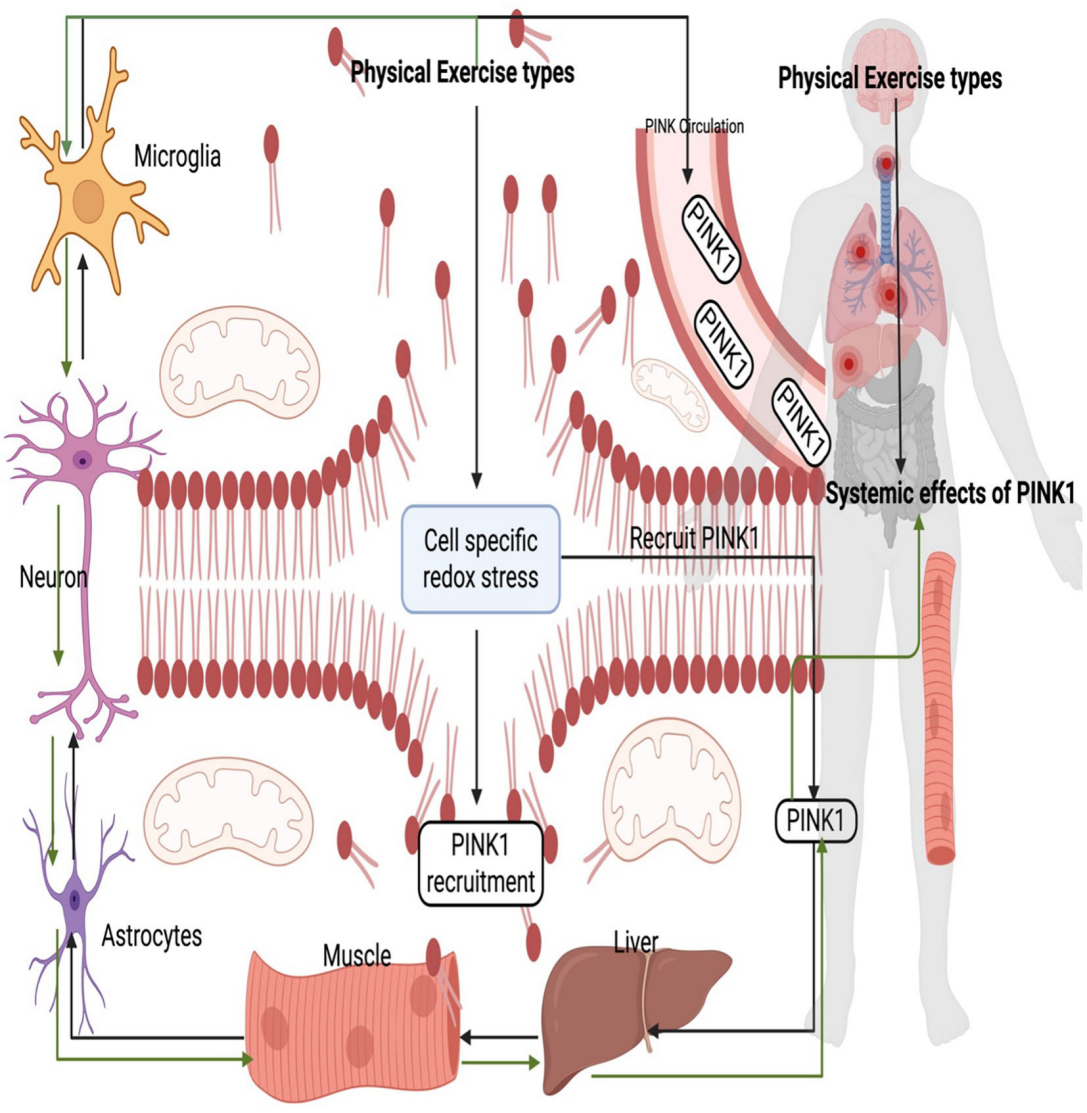
Different types of exercise stimulate diverse cell populations to trigger a systemic effect of PINK1 by activating cell-specific redox stress in skeletal muscle, liver, astrocytes, neurons, and glial cells. This process systematically increases PINK1 activation to enhance mitochondrial quality across various tissues.

Exercise types attract distinct neural and glial populations, and this cell-specific stress pattern may modulate PINK1 expression and PINK1/Parkin-dependent mitophagy in the brain (**Figure 3**). Exercise-induced redox shifts, the NAD^+^/NADH ratio, and downstream deacetylase signaling that converge on FOXO3, histones, and mitochondrial enzymes may be the primary drivers of these effects (Chen et al., 2022; Poljsak and Milisav, 2016). For instance, 8 weeks of running exercise (5 days/week) promotes region-specific mitochondrial remodeling in the hippocampus by elevating PINK1 and Parkin levels, thereby enhancing neuronal resilience (Nikolaidis et al., 2012). However, the temporal cortex showed a blunted response to PINK1, highlighting regional differences in signaling sensitivity to exercise. Simultaneously, endurance training upregulates Tricarboxylic acid cycle (TCA) enzymes, including citrate synthase, aconitase, and α-ketoglutarate dehydrogenase, resulting in reinforcing mitochondrial bioenergetic capacity in neurons and glia [71-74]. However, the mechanism by which PINK1 directly interacts with these enzymes under exercise conditions remains unknown. Exercise-induced upregulation of TCA cycle enzymes may reciprocally interact with PINK1 to drive mitochondrial metabolic reprogramming toward lipid synthesis, achieved by modulating aconitase activity to promote citrate accumulation while preserving alpha-ketoglutarate dehydrogenase (α-KGDH) function. This mechanism is supported by Bus et al. (2019), who demonstrated that PINK1 regulates dopamine and lipid metabolism in neuronal mitochondria (Bus et al., 2020), although their study did not examine the effects of exercise. Complementary studies documenting exercise effects on PINK1 and TCA cycle enzymes reinforce this pathway (Kang et al., 2025; Navarro et al., 2004; Marques-Aleixo et al., 2015; Bus et al., 2019), highlighting PINK1’s broader function in enhancing neuronal energy homeostasis, beyond mitophagy induction, to maintain metabolic balance. Long-term aerobic exercise increases PINK1 levels in the cortex, promoting efficient removal of dysfunctional mitochondria and improving bioenergetics after 12 weeks of training (Bus et al., 2019). Mechanistically, sustained exercise training maintains an elevated NAD^+^/NADH ratio and SIRT1/SIRT3 activity. This scenario can prolong FOXO3 deacetylation and enhance a transcriptional program that couples mitochondrial biogenesis with selective mitophagy to optimize mitochondrial quality in brain cells.

## 7 Effect of exercise on PINK1-regulating pathways

**Figure 4:**
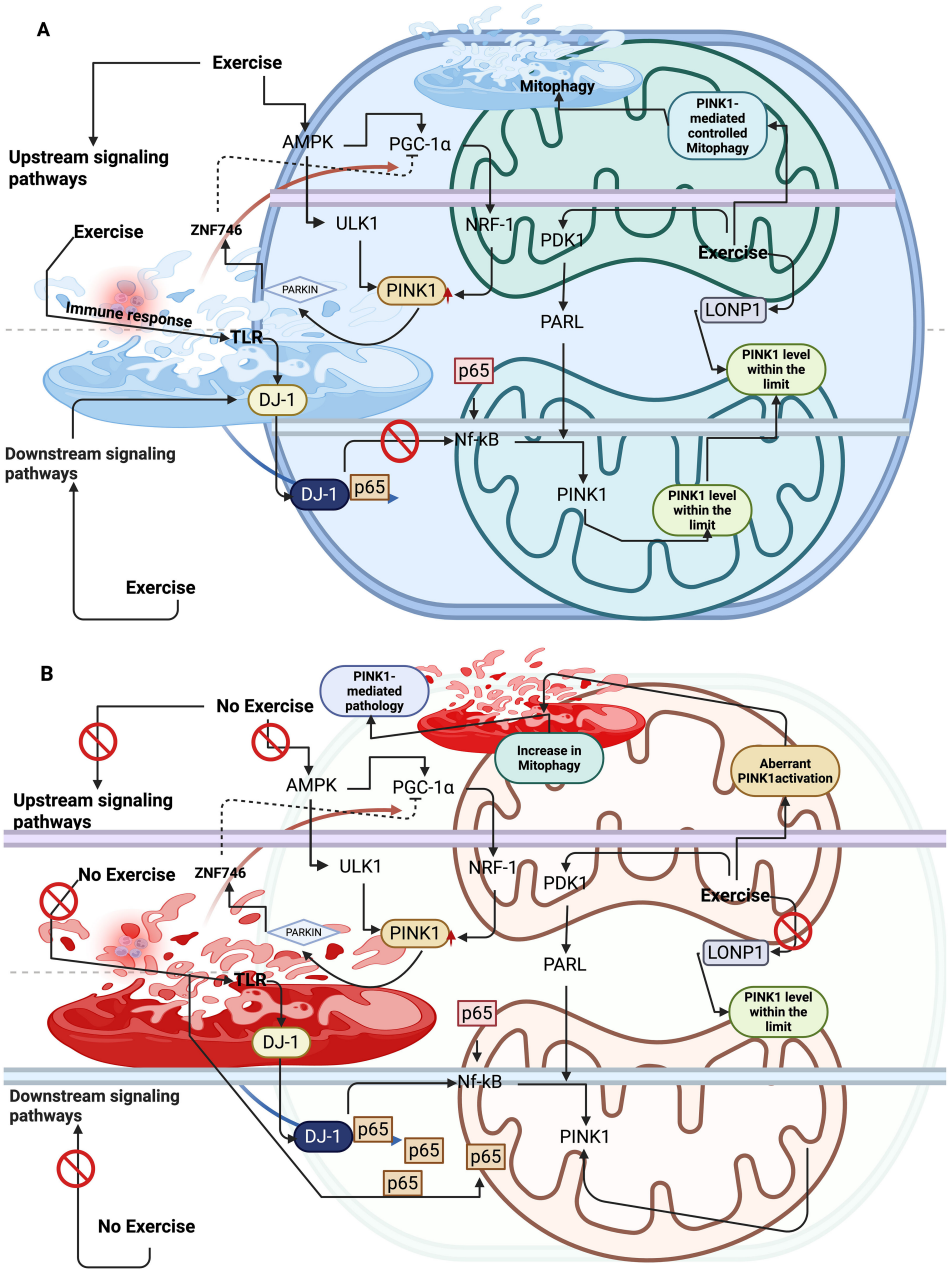
Physical exercise regulates PINK1-mediated pathways. **(A)** Upstream pathways of PINK1, such as AMPK and PGC-1α, activate Nrf2 to promote PINK transcription during exercise. Exercise exacerbates the immune response, activating downstream pathways such as DJ-1 via TLR signaling, which, in turn, inhibits NF-κB to regulate PINK1. Also, exercise-induced LONP1 keeps PINK1 within limits and promotes controlled mitophagy. **(B)** Both upstream and downstream pathways of PINK1 are inhibited under non-exercise conditions, leading to aberrant PINK1 activation and accumulation, as well as PINK1-mediated pathology.

Physical exercise modulates both upstream and downstream PINK1 signaling by preserving mitochondrial integrity and stabilizing the membrane potential. As a result, selective mitophagy occurs only in genuinely impaired mitochondria. For example, loss of the DJ-1 blocks mitophagy by impairing mitochondrial recruitment of optineurin in human fibroblasts and neurons (Imberechts et al., 2022). Physical exercise appears to upregulate DJ-1 via RAGE and TLR signaling to maintain mitochondrial function. This can enhance cognitive and motor functions. Two exercise-mediated mechanisms may regulate DJ-1–PINK1 signaling. First, as mentioned above, exercise-induced oxidation of DJ-1 at Cys106 can enhance its expression (Miyama et al., 2011; Day et al., 2024), allowing DJ-1 to sequester p65 in the cytoplasm, where it represses NF-κB activity and thereby reduces PINK1 expression (**Figure 4**). An increase in DJ-1 has been shown to facilitate the dissociation of p65 from NF-κB inhibitor α, which protects dopaminergic neurons in Parkinson’s disease (Lin et al., 2021). Although this study did not directly examine exercise or PINK1, other reports describing the causal links among exercise, DJ-1 upregulation, NF-κB signaling, and PINK1 suggest that exercise may regulate this mechanism (Shi et al., 2015; Liu and Chang, 2018). Second, exercise-induced immune perturbation may stimulate Toll-like receptors (TLR) pathways that upregulate DJ-1, which, in parallel, supports or acts downstream of PINK1 to efficiently mediate PINK1/Parkin-mediated mitophagy (Moghazy et al., 2026). Mitochondrial proteostasis maintains PINK1 at low levels on healthy mitochondria to prevent unnecessary accumulation of PINK1 and increase PINK1’s damage-sensing activity. Lon protease 1 (LONP1) is a mitochondrial matrix protease that regulates mitochondrial proteostasis. Several studies have reported that exercise upregulates LONP1 in metabolically active tissues (Tang et al., 2025; Zanini et al., 2023; Xu et al., 2022), which may help exercise maintain PINK1 within limits and selectively target damaged mitochondria. PARL is a mitochondrial inner-membrane rhomboid protease that mediates PINK1 cleavage and degradation (Sekine et al., 2012; Shi and McQuibban, 2017). Although the direct effects of physical exercise on PARL have not been reported, aerobic training increases PDK2 expression, which, in turn, can modulate PARL activity and thus support mitochondrial quality and metabolic function by regulating PINK1 cleavage and degradation. Indirect signaling pathways such as AMPK and PGC-1α can modulate PINK1 activation by optimizing cellular energy supply and mitochondrial quality (Thirupathi and de Souza, 2017). Exercise-induced mitophagy may therefore arise from coordinated activation of AMPK and the PINK1/Parkin pathway, although each pathway can also initiate mitophagy independently. Notably, exercise performance and overall physical fitness appear largely unaffected by PINK1, whereas AMPK has a measurable effect. For example, exercise-induced AMPK activation stimulates ULK1 (Campos et al., 2023; Laker et al., 2017), which can act as an upstream signaling pathway for PINK1 or operate in parallel with the PINK1/Parkin pathway to enhance mitophagy in neurons and other brain cells. Mechanistically, exercise-induced activation of ULK1 can rapidly phosphorylate Parkin at Ser108, priming Parkin for subsequent PINK1-dependent phosphorylation at Ser65, hence, enhancing PINK1/Parkin-mediated mitophagy (Seabright and Lai, 2020). Also, exercise-activated PGC-1α may be regulated by a feedback loop involving PINK1-Parkin via ZNF746, a PGC-1α repressor. The increase in PARIS/ZNF746 creates a detrimental loop in this mechanism that disrupts mitochondrial biogenesis (Antico et al., 2021; Pickrell and Youle, 2015). However, the direct effect of exercise on ZNF746 measurement is limited. Drawing on these molecular regulatory mechanisms, the physiological expression of PINK1 is not uniform across the body; rather, it exhibits distinct, tissue-specific activation thresholds in response to exercise load.

## 8 Magnitude of exercise-induced PINK1 activation in different tissues

Exercise-induced activation of PINK1 is generally moderate and depends on duration, modality, and the specific tissue examined, rather than being maximal or continuous. For example, 10 weeks of progressive-load swimming and 6 weeks of resistance training both increase PINK1 in the cardiac muscle, whereas resistance training produces the maximum increase in PINK1 levels (Nambu et al., 2021). Additionally, 9 weeks of aerobic training increases PINK1 in heart failure models (Jia et al., 2025). In skeletal muscle, chronic protocols, such as 5 weeks of climbing, 12 weeks of treadmill running, 4 weeks of moderate swimming, and downhill running consistently elevate PINK1, whereas three consecutive days of acute exercise do not increase PINK1, indicating that sustained loading is required to engage PINK1-mediated mitophagy (Zhao et al., 2023; Yu et al., 2025). In the brain, 12 weeks of treadmill exercise enhances PINK1/Parkin-dependent mitophagy in the hippocampus, highlighting the importance of prolonged training stimuli (Zhao et al., 2023). These results indicate that the PINK1 response to exercise varies by tissue. Both endurance and resistance paradigms robustly upregulate PINK1, whereas skeletal muscle requires adequate volume and mechanical strain; long-term aerobic training is required to elevate PINK1 in hippocampal neurons (Zhao et al., 2023; Jia et al., 2025). Nevertheless, acute exercise often fails to increase PINK1, underscoring that only certain tissues and protocols effectively recruit this mitophagy pathway. Other individual factors further modulate PINK1 dynamics. For example, 4 weeks of voluntary wheel running reduces PINK1 levels, suggesting that aging may blunt, or even reverse, exercise-induced PINK1 upregulation (Chen et al., 2018) The timing of exercise can also influence PINK1 expression, as evidenced by a 28-day resistance exercise intervention, which increases PINK1 in men from baseline to 12 h, whereas a single sprint session in highly trained endurance athletes does not alter PINK1 within 3 h, indicating that gender, age, and prior training status shape the temporal profile and magnitude of the PINK1 response (**Table 1**) (Luk et al., 2022; Yu et al., 2025).

**Table 1:** Demographic modulators of PINK1 activation: a framework for personalized exercise prescription.

Demographic factor	Basal PINK1/Mitophagy status	Exercise-induced PINK1 response	Proposed personalized exercise framework	Key supporting evidence
Aging (Older Adults >65 yrs)	Gradual decline of PINK1/Parkin expression in aging trggers basal oxidative stress and damaged mitochondria	Sustained stimulus like exercise is needed to overcome age-related PINK1 threshold.	Chronic low to moderate aerobic training along with pharmacological agents may lower the activation threshold.	Age-dependent loss of PINK1/Parkin rises mitophagy (Cornelissen et al., 2018); exercise may reverse this (Lin and Wen, 2026)
Young/Healthy Adults	PINK1 expression are in steady-state; resulting in efficient baseline mitochondrial quality control.	PINK1 activation to moderate-to-vigorous acute metabolic stress; clear temporal activation window.	Moderate-to-vigorous aerobic exercise (60-75% HRmax) and HIIT protocols. No pharmacological synergy required.	Established physiological baselines (Drake et al., 2019).
Female	Estrogen improves mitochondrial quality control via SIRT1/PINK1 axes;	Exercise induced estrogen sensitize PINK1 activation to facilitate rapid mitochondrial clearance.	Standard aerobic and resistance training. Protocols can be periodized alongside the menstrual cycle to leverage natural estrogen peaks.	Estrogen regulates Rab9 and PINK1-dependent mitophagy (Sasaki et al., 2021); sex differences are crucial factor for mitochondrial adaptation (Smith et al., 2013)
Female and male	The inolvement of PINK1 in males are crucial for stress management than female	Exercise induced PINK1 activation is more crucial in males in managing stress-induced mitophagy compare to females	Chronic exercise with three times per week may alliviate the stress management in both genders	Sex differenrnces affects the behavioural responses through PINK1 mediated mitophagy (Feehan and Kritzer, 2025)
Sedentary / Untrained Status	Low basal mitochondrial turnover; diminished endogenous antioxidant enzyme such as SOD2.	Different exercise types triggers PINK1/PArkin stabilization and enhance antioxidant effect. Resistance type of exercise has the best effect on PINK activation	Begin with low intensity aerobic exercise to build basic mitochondrial network and antioxidant capacity before introducing high intensity aerobic or resistance types to improve PINK1	Untrainied fails to stabilize PINK1 (Drake et al., 2019; Li et al., 2021).
Trained status	Chronic elevation of basal PINK1	Training status rescue PINK1/Parkin-mediated mitophagy	Requires significant progressive overload to keep maintaining basal PINK1 (e.g., exhaustive HIIT or prolonged endurance)	Training adaptations chronically upregulate mitophagy machinery, altering acute stress thresholds (Laker et al., 2017; Zhao et al., 2023).

## 9 Can exercise synergize with pharmacological agents to enhance PINK1-dependent mitophagy?

Combining physical exercise with pharmacological and natural agents can serve as complementary strategies to activate PINK1. For example, regular exercise helps maintain mitochondrial quality and sensitizes the PINK1/Parkin system, thus requiring lower doses of these drugs to activate PINK1 and trigger selective mitophagy rather than catastrophic cell death. For example, exercise-induced mild mitochondrial stress, via a transient increase in ROS production and fluctuations in membrane potential, activates AMPK–PGC-1α, Nrf2, and SIRT1/SIRT3–FOXO3a (Pilegaard et al., 2003), which, in turn, increases PINK1/Parkin expression and LC3-dependent mitophagy. Also, these same transcriptional and redox pathways are targeted by natural compounds, including resveratrol, urolithin A, β-asarone, cyanidin-3-O-glucoside, and ginsenosides, which increase SIRT1/FOXO3a signaling and LC3II formation while upregulating PINK1/Parkin-mediated mitophagy in models of neurodegeneration and metabolic disease (Shao et al., 2026). Thus, regular exercise training may synergistically act with these natural modulators to target selective mitophagy via improving PINK1. Additionally, small molecules such as MTK458 and FB231 may target mitochondrial toxins to prevent their catastrophic effects and avoid triggering rapid cell death; otherwise, these toxins can robustly induce cell death before PINK1 fully activates (Rosencrans et al., 2025). The use of antimycin, which specifically inhibits complex III of the ETC and collapses the mitochondrial membrane to facilitate PINK1 import, while the use of oligomycin, which disrupts mitochondrial ATP synthase to promote PINK1 accumulation by causing metabolic stress and altering the membrane potential (Quinlan et al., 2011). Despite clear mechanistic overlap between exercise-induced signaling and these pharmacologic or natural modulators, no studies have directly examined their combined use with exercise to enhance PINK1 activation *in vivo*.

## 10 Neglected effects of exercise on PINK1 regulation

While all forms of exercise induce minimal cellular and tissue damage, certain modalities inflict greater cellular damage. Nevertheless, the mitochondrial consequences of this damage, particularly within the context of PINK1-mediated mitophagy, remain underexplored. Most importantly, the magnitude of exercise-induced muscle and non-muscle damage, including ROS, and its effects on PINK1 activation remain poorly defined and warrant comprehensive investigation. For example, most studies that reported the effect of exercise on ROS markers, such as MDA and F2-isoprostanes from blood specimens immediately post exercise for assessing the peak level of oxidative stress, but highlighting blood-centric studies may overlook the nuances of PINK1 responses in other cells and tissues, as mitochondrial response occurs only later, 3–6 hours during recovery in the skeletal muscle and liver (Singh et al., 2024; McCoin et al., 2022). Also, tissue-specific strategies for exercise-induced PINK1 activation and clearance reveal significant research gaps. For example, muscle models in exercised conditions systematically undervalue PINK1’s role, while neural and hepatic models tend to overestimate it. This disparity arises from a muscle-centric bias that dominates exercise physiology, in which PINK1-independent mitophagy predominates. In contrast, neural and hepatic models often reported PINK1’s pathological effects, portraying exercise as a PINK1 inducer and exaggerating its physiological effects. Notably, most studies failed to account for basal ROS levels and redox potential, as these factors vary across tissues. For instance, skeletal muscle has higher basal ROS levels than neural and hepatic tissues, while the liver has a more oxidized redox potential and the brain has a more reduced redox potential, with lower GSH/GSSG ratios, which often sensitize PINK1 even at milder exercise conditions and lower intensities than in skeletal muscle (Gao et al., 2020). Considering all these factors can help address integrated deficits in multi-tissue research under controlled exercise, and comprehensive cross-tissue studies can fully unlock exercise as a potential regulator of PINK across physiological and pathological states.

## 11 The tissue-specific thresholds for PINK1 activation

The threshold for PINK1 activation varies among different tissues, and characterizing this heterogeneity is critical for designing exercise-based interventions for PD-like conditions. For example, muscle and liver tissues possess higher PINK1 thresholds, requiring substantial physiological stimuli. In this context, HIIT or vigorous resistance exercise can induce ROS, which maintains redox buffering capacity and stabilizes PINK1 without causing oxidative stress. Supporting this, Drake et al. (2019) reported that acute exercise with low to moderate intensity did not trigger PINK1-mediated mitophagy in skeletal muscle, indicating that alternative pathways, such as ULK1 via AMPK-dependent phosphorylation at Ser555, may mediate mitophagy. Also, current evidence indicates that PINK1/Parkin activation by exercise is largely inferred from measurements of upstream regulators rather than measured directly, suggesting that exercise can act as a systemic stressor that induces selective mitophagy via several redox signaling pathways, including AMPK. In addition, PINK1 activation and its subsequent stabilization during exercise may reflect downstream consequences of exercise intensity that can surpass the PINK1 threshold. For example, Khoramipour et al. reported that HIIT protocols upregulated PINK1 and PINK1-dependent mitophagy in breast cancer cells, underscoring the importance of exercise intensity in overcoming tissue-specific activation thresholds when targeting PINK1 (Khoramipour et al., 2024). Nevertheless, the contribution of other mitophagy pathways under basal conditions, when PINK1 activation is insufficient, remains unclear. A recent study shows that PINK1 knockout under basal conditions does not significantly reduce mitophagy in most tissues, suggesting the involvement of alternative mitochondrial quality control mechanisms. However, loss of PINK1 in oxidative muscle tissue reduces basal mitophagy (Drake et al., 2019), indicating that injury or ROS-induced stress may be required to activate PINK1-dependent mitophagy. Moreover, elucidating tissue-specific roles of PINK1-mediated mitophagy could reveal new therapeutic strategies for PD-like conditions. For example, skeletal muscle, which exhibits higher basal ROS production during exercise and increased antioxidant capacity, may serve as a valuable model for investigating PINK1-mediated mitophagy, as recent evidence shows that PINK1 deficiency in oxidative muscle reduces basal mitophagy (Singh et al., 2024). However, exercise modalities that induce minimal oxidative stress may sufficiently activate PINK1-mediated mitophagy in skeletal muscle, and whether this effect extends to the brain remains to be determined. Additionally, Murphy et al. reported that PD is characterized by muscle-specific pathologies, including mitochondrial dysfunction (Murphy and Lynch, 2023), suggesting shared mechanisms between skeletal muscle and brain. PINK1 activation could therefore function as an early biomarker for PD onset. Brain regions exhibit stricter redox homeostasis and lower tolerance for oxidative disturbances, so modest ROS elevations may initiate PINK1-mediated mitophagy but also risk oxidative damage if excessive. Thus, moderate-intensity exercise of longer duration may be safer and more effective for promoting PINK1-dependent mitophagy in the brain.

In addition to exercise intensity and tissue-specific thresholds for PINK1 activation, understanding the temporal dynamics of PINK1 activation is essential for optimizing its clinical application. Laker et al. reported that mitophagy in skeletal muscle peaks six hours after acute treadmill exercise, primarily through AMPK and Ulk1, following a transient increase in mitochondrial oxidative stress between three and twelve hours (Laker et al., 2017). This mitophagy peak depends on AMPK-ULK1 signaling rather than PINK1, indicating a key mechanistic distinction between physiological and pathological mitochondrial quality control. Furthermore, no PINK1 stabilization occurs within the 24-hour recovery period (Drake et al., 2019). However, HIIT-induced severe redox collapse or energetic stress can disrupt membrane potential, leading to PINK1 stabilization and Parkin recruitment within three to six hours post-exercise. Chronic exercise may also elevate basal PINK1 levels, supporting sustained mitophagy. Whether similar effects occur in the brain, as observed in skeletal muscle and liver, or whether the brain requires a longer recovery phase for mitochondrial clearance under strict redox regulation, remains unclear. Defining these temporal windows will inform optimal recovery periods and exercise timing and may facilitate synergistic interventions with pharmacological agents for PD-like conditions. Additionally, this knowledge can guide the timing of muscle biopsies to leverage peripheral PINK1 activation as an early biomarker for PD. Ultimately, elucidating the temporal kinetics of PINK1 will advance exercise-induced mitophagy from a basic biological phenomenon to a personalized therapeutic strategy for the management of neurodegenerative diseases.

## 12 PINK1’s role in adipose tissue

Considering the role of PINK1-mediated mitophagy in adipose tissue could clearly link PINK1 to regulating lipid metabolism and, in turn, energy homeostasis. Primarily, PINK1/Parkin recruitment by PNPLA7 inhibits the browning of adipose tissue for mitophagy in adipose tissue (Ji et al., 2020). Although the role of exercise in regulating PNPLA7 and further PINK1/Parkin recruitment remains unexplored, studies have shown that metabolically active tissues such as skeletal muscle, adipose tissue, and the liver are highly expressed for PNPLA7, which dynamically responds to energy availability and metabolic stress (Ji et al., 2020). Notably, PNPLA7 is activated during fasting, its activity is altered by cAMP signaling pathways, and this pathway is more sensitive to exercise. Therefore, it is highly possible that exercise can interfere with the activation of PNPLA7 and further PINK1/Parkin-mediated mitochondrial clearance to preserve mitochondrial networks that are crucial for browning of white adipose tissue. So, understanding the crosstalk between PNPLA7 and PINK1/Parkin, and the relationship to mitochondrial quality and energy homeostasis, may shed light on how exercise systematically regulates metabolic health. Furthermore, contextualizing exercise-induced PINK1’s role in metabolic diseases may further highlight the translational relevance of exercise-induced mitochondrial quality control. For example, higher metabolic stress and disrupted energy homeostasis may trigger insulin resistance and altered metabolism. In this scenario, exercise-mediated PINK1/Parkin-mediated mitophagy could serve as a compensatory mechanism to promote the effective clearance of dysfunctional mitochondria and improve metabolic health.

## 13 Specific combination protocols as a roadmap for PINK1-mediated clinical trials

Maximizing the therapeutic potential of PINK1-mediated mitophagy requires a focus on synergistic or multimodal strategies that combine pharmaceutical agents with targeted interventions. This approach is particularly relevant because low-dose exercise alone often does not fully stabilize PINK1, and high-intensity interval training protocols are frequently unsuitable for elderly individuals with neurodegenerative disorders. In this context, combining mitophagy enhancers such as resveratrol, a natural polyphenol, with aerobic training may reduce the threshold for PINK1 activation. Resveratrol induces PINK1 transcription, while aerobic exercise generates mild metabolic and oxidative stress that sustains PINK1 activation, thereby facilitating efficient mitophagy (Sebori et al., 2018). Additional mitophagy enhancers, including urolithin and spermidine, when used in conjunction with exercise, may safely promote mitophagy in neurodegenerative conditions (Borsky et al., 2025). Furthermore, redox modulators and NAD precursors, such as nicotinamide riboside and nicotinamide mononucleotide, activate the Sirt1/AMPK axis and stabilize PINK1 (Li et al., 2023). Other modulators, including kinetin and MTK458, may sensitize PINK1 activity (Gan et al., 2024). The integration of these compounds is expected to advance PINK1-mediated therapies from both *in vitro* and *in vivo* models toward disease-modifying treatments for various neurodegenerative disorders.

## 14 Tissue model biases for guide exercise precision strategies

Current mitophagy research exhibits a significant interpretive gap due to tissue-model biases, as most studies have focused predominantly on muscle tissue (Rahman et al., 2025; Drake and Yan, 2017). Consequently, there is a tendency to overemphasize neural mechanisms, particularly regarding PINK1-mediated mitophagy in the brain. For instance, frequent ROS flux resulting from muscle contraction leads to a robust antioxidant system in skeletal muscle (Powers et al., 2024). In contrast, neurons and other brain cells possess limited total antioxidant capacity, rendering them more susceptible to oxidative stress and related damage (Lee et al., 2020). Therefore, it is inappropriate to assume that exercise protocols activating PINK1 in skeletal muscle will yield equivalent effects in brain cells. Moreover, existing studies on PINK1 activation by various exercise protocols have not adequately investigated it in different tissues. Addressing this limitation requires a shift from single-organ to cross-tissue research approaches. Understanding cross-tissue standardization requires quantifying differences in basal ROS levels among organs, as PINK1 activation and its threshold are regulated by redox flux (Gan et al., 2024). Thus, mapping baseline ROS production and total antioxidant capacity across tissues is essential. Developing a ‘redox index’ based on ROS quantification across different tissues could facilitate calibration of exercise intensities to activate PINK1 in both brain cells and oxidative muscle fibers. Additionally, employing dual experimental designs that track real-time ROS fluctuations alongside mitophagy in various tissues during exercise may provide more comprehensive insights. Assessing the effects of exercise intensity on local tissue-specific ROS formation could further bridge the interpretive gap between muscle and brain. Ultimately, quantifying basal ROS across multiple tissues may inform precision exercise strategies, supporting safe therapeutic interventions to promote PINK1-mediated neuroprotection in neurodegenerative diseases and other mitochondriopathies.

## 15 Distinguishing established evidence from hypothesized mechanisms

This review combines strong evidence on PINK1 mechanisms with new theoretical insights. It is well established in the literature that physical exercise influences PINK1/Parkin activity, primarily based on *in vitro* studies and observed in peripheral tissues such as skeletal muscle. In these tissues, researchers have clearly documented how redox kinetics affect physiological responses, cysteine oxidative states, and clearance pathways. Using this solid evidence as a fundamental concept, we propose a working model for how these exact mechanisms might translate to the CNS. Specifically, we suggest that physical exercise could block PARL-mediated over-cleavage in the brain and interrupt the neuronal “vicious cycle” of oxidative stress. While this idea is highly plausible, it remains a hypothesized framework. Furthermore, this proposed mechanism is strongly supported by cellular redox dynamics data from peripheral tissues, and PINK1-mediated neuroprotective pathways still need to be validated directly in CNS models when interpreting these data. By clearly separating what is already known in peripheral tissues from what we hypothesize occurs in the brain, we hope this review guides future research toward the use of targeted, exercise-induced mitophagy for clinical neuroprotection.

## 16 Conclusions and future directions

This review elucidates how physical exercise activates and recruits PINK1 through various mechanisms, positioning exercise as a potent effector across tissues. Despite striking discrepancies in methodological details and tissue-specific responses, diverse exercise modalities consistently associate with PINK1 activation, underscoring its therapeutic promise. With appropriate changes to the exercise protocols, it can trigger PINK1 in all tissues and organs. This can promote selective mitophagy. Exercise-induced ROS can be the primary driver of this effect through various oxidative processes in amino acids such as cysteine, methionine, and tyrosine, all of which, during oxidation, can cause local disruption within helical regions and shift the helix-coil balance, destabilizing the PINK1 structure. Nevertheless, understanding the transient systemic elevation of this effect during or post-exercise further strengthens the role of exercise in activating PINK1. Notably, exercise disrupts the vicious cycle of PINK1 oxidation by regulating the PINK1/CHCHD4/GFER disulfide relay system without overwhelming the IMS relay. Other major pathways, such as APE1, Nrf2, FOXO3, and DNAJB1/HSP40, are activated or suppressed post-exercise, and these pathways have binding sites in the PINK1 promoter regions, indicating their role in PINK1 transcription. Beyond its role in activating PINK1 for selective mitophagy, exercise modulates TCA cycle enzymes to rewire mitochondrial metabolism, underscoring PINK1’s broader physiological functions. Despite these examples highlighting the role of exercise in PINK1 activation, the complexity of PINK1 expression across tissues, along with other factors such as age, gender, and an individual’s training status, may challenge current tools that fail to capture the local-to-systemic effects of exercise-induced PINK1 activation. These gaps hinder understanding of exercise-mediated PINK1 and its therapeutic translation. Applying integrative approaches to these factors may shed light on exercise-PINK1 interactions, enabling precise interventions for mitochondrial health.

## References

[B1] Anderson C. A. Kovar D. R. Gardel M. L. Winkelman J. D. (2021). Lim domain proteins in cell mechanobiology. Cytoskeleton. (Hoboken) 78, 303–311. doi: 10.1002/cm.21665 34028199 PMC8603407

[B2] Antico O. Ordureau A. Stevens M. Singh F. Nirujogi R. S. Gierlinski M. . (2021). Global ubiquitylation analysis of mitochondria in primary neurons identifies endogenous Parkin targets following activation of PINK1. Sci. Adv. 7, eabj0722. doi: 10.1126/sciadv.abj0722 34767452 PMC8589319

[B3] Antunes F. Brito P. M. (2017). Quantitative biology of hydrogen peroxide signaling. Redox Biol. 13, 1–7. doi: 10.1016/j.redox.2017.04.039 28528123 PMC5436100

[B4] Arena G. Modjtahedi N. Kruger R. (2021). Exploring the contribution of the mitochondrial disulfide relay system to Parkinson's disease: the PINK1/CHCHD4 interplay. Neural Regen. Res. 16, 2222–2224. doi: 10.4103/1673-5374.310680 33818502 PMC8354137

[B5] Barodia S. K. Creed R. B. Goldberg M. S. (2017). Parkin and PINK1 functions in oxidative stress and neurodegeneration. Brain Res. Bull. 133, 51–59. doi: 10.1016/j.brainresbull.2016.12.004 28017782 PMC5718625

[B6] Basak B. Holzbaur E. L. F. (2025). Mitophagy in neurons: mechanisms regulating mitochondrial turnover and neuronal homeostasis. J. Mol. Biol. 437, 169161. doi: 10.1016/j.jmb.2025.169161 40268233

[B7] Bento-Pereira C. Dinkova-Kostova A. T. (2021). Activation of transcription factor Nrf2 to counteract mitochondrial dysfunction in Parkinson's disease. Med. Res. Rev. 41, 785–802. doi: 10.1002/med.21741 32681666

[B11] Borsky P. Holmannova D. Soukup O. Fiala Z. Maresova T. Hanzlova M. . (2025). Distinct roles of urolithin A and spermidine in mitophagy and autophagy: implications for dietary supplementation. Nutr. Res. Rev. 17, 39:e8. doi: 10.1017/s0954422425100292 41404767

[B8] Bueno M. Brands J. Voltz L. Fiedler K. Mays B. St Croix C. . (2018). Atf3 represses PINK1 gene transcription in lung epithelial cells to control mitochondrial homeostasis. Aging Cell 17, e12720. doi: 10.1111/acel.12720 29363258 PMC5847866

[B10] Bus C. Geisler S. Feldkaemper M. Flores-Romero H. Schaedler A. Zittlau K. . (2019). Pink1 regulates dopamine and lipids at mitochondria to maintain synapses and neuronal function. bioRxiv. doi: 10.1101/814343. Preprint. 38621210

[B9] Bus C. Zizmare L. Feldkaemper M. Geisler S. Zarani M. Schaedler A. . (2020). Human dopaminergic neurons lacking PINK1 exhibit disrupted dopamine metabolism related to vitamin B6 co-factors. iScience 23, 101797. doi: 10.1016/j.isci.2020.101797 33299968 PMC7702004

[B12] Callegari S. Kirk N. S. Gan Z. Y. Dite T. Cobbold S. A. Leis A. . (2025). Structure of human PINK1 at a mitochondrial TOM-VDAC array. Science 388, 303–310. doi: 10.1126/science.adu6445 40080546

[B13] Campos J. C. Marchesi Bozi L. H. Krum B. Grassmann Bechara L. R. Ferreira N. D. Arini G. S. . (2023). Exercise preserves physical fitness during aging through AMPK and mitochondrial dynamics. Proc. Natl. Acad. Sci. U.S.A. 120, e2204750120. doi: 10.1073/pnas.2204750120 36595699 PMC9926278

[B14] Chen C. C. W. Erlich A. T. Crilly M. J. Hood D. A. (2018). Parkin is required for exercise-induced mitophagy in muscle: impact of aging. Am. J. Physiol. Endocrinol. Metab. 315, E404–E415. doi: 10.1152/ajpendo.00391.2017 29812989

[B15] Chen Y. Luo Z. Sun Y. Li F. Han Z. Qi B. . (2022). Exercise improves choroid plexus epithelial cells metabolism to prevent glial cell-associated neurodegeneration. Front. Pharmacol. 13, 1010785. doi: 10.3389/fphar.2022.1010785 36188600 PMC9523215

[B16] Cornelissen T. Vilain S. Vints K. Gounko N. Verstreken P. Vandenberghe W. (2018). Deficiency of parkin and PINK1 impairs age-dependent mitophagy in Drosophila. Elife 7, e35878. doi: 10.7554/elife.35878 29809156 PMC6008047

[B17] Das Banerjee T. Dagda R. Y. Dagda M. Chu C. T. Rice M. Vazquez-Mayorga E. . (2017). Pink1 regulates mitochondrial trafficking in dendrites of cortical neurons through mitochondrial PKA. J. Neurochem. 142, 545–559. doi: 10.1111/jnc.14080 28556983 PMC5554084

[B18] Day N. J. Kelly S. S. Lui L. Y. Mansfield T. A. Gaffrey M. J. Trejo J. B. . (2024). Signatures of cysteine oxidation on muscle structural and contractile proteins are associated with physical performance and muscle function in older adults: Study of Muscle, Mobility and Aging (SOMMA). Aging Cell 23, e14094. doi: 10.1111/acel.14094 38332629 PMC11166363

[B19] Deas E. Plun-Favreau H. Gandhi S. Desmond H. Kjaer S. Loh S. H. . (2011). Pink1 cleavage at position A103 by the mitochondrial protease PARL. Hum. Mol. Genet. 20, 867–879. doi: 10.1093/hmg/ddq526 21138942 PMC3033179

[B20] Díaz-Castro F. Tuñón-Suárez M. Rivera P. Botella J. Cancino J. Figueroa A. M. . (2024). A single bout of resistance exercise triggers mitophagy, potentially involving the ejection of mitochondria in human skeletal muscle. Acta Physiol. (Oxf) 240, e14203. doi: 10.1111/apha.14203 39023008

[B22] Drake J. C. Laker R. C. Wilson R. J. Zhang M. Yan Z. (2019). Exercise-induced mitophagy in skeletal muscle occurs in the absence of stabilization of Pink1 on mitochondria. Cell. Cycle 18, 1–6. doi: 10.1080/15384101.2018.1559556 30558471 PMC6343730

[B21] Drake J. C. Yan Z. (2017). Mitophagy in maintaining skeletal muscle mitochondrial proteostasis and metabolic health with ageing. J. Physiol. 595, 6391–6399. doi: 10.1113/JP274337 28795394 PMC5638883

[B23] Duan H. Hu J. Yan H. Zhou S. (2014). Moderate exercise decreases the expression of PTGS2 and NF-κB in skeletal muscle of ovariectomized rats. BioMed. Res. Int. 2014, 169245. doi: 10.1155/2014/169245

[B24] Essers M. A. Weijzen S. de Vries-Smits A. M. Saarloos I. de Ruiter N. D. Bos J. L. . (2004). Foxo transcription factor activation by oxidative stress mediated by the small GTPase Ral and JNK. EMBO J. 23, 4802–4812. doi: 10.1038/sj.emboj.7600475 15538382 PMC535088

[B26] Feehan S. M. Kritzer M. F. (2025). Sex differences in behavioral measures of anxiety in a recessive gene knockout (Pink1-/-) rat model of Parkinson's disease. Front. Behav. Neurosci. 19, 1646733. doi: 10.3389/fnbeh.2025.1646733 40963536 PMC12436465

[B25] Fernández-Verdejo R. Vanwynsberghe A. M. Essaghir A. Demoulin J. B. Hai T. Deldicque L. . (2017). Activating transcription factor 3 attenuates chemokine and cytokine expression in mouse skeletal muscle after exercise and facilitates molecular adaptation to endurance training. FASEB J. 31, 840–851. doi: 10.1096/fj.201600868R 27856557

[B27] Furrer R. Handschin C. (2024). Molecular aspects of the exercise response and training adaptation in skeletal muscle. Free Radic. Biol. Med. 223, 53–68. doi: 10.1016/j.freeradbiomed.2024.08.015 39059515 PMC7617583

[B37] Gan Z. Y. Komander D. Callegari S. (2024). Reassessing kinetin's effect on PINK1 and mitophagy. Autophagy. 20, 2596–2597. doi: 10.1080/15548627.2024.2395144 39342462 PMC11572244

[B30] Gao Q. Tian R. Han H. You H. Fan L. Li R. . (2022). Pink1-mediated Drp1S616 phosphorylation modulates synaptic development and plasticity via promoting mitochondrial fission. Signal. Transduction Targeting Ther. 7, 103. doi: 10.1038/s41392-022-00933-z 35422062 PMC9010405

[B28] Gao B. Wang L. Gong J. Zhu Z. Liu Q. Yuan H. . (2025). The interplay between physical exercise and autophagy signaling in brain health, neurodegenerative diseases and aging. Front. Aging Neurosci. 17, 1579208. doi: 10.3389/fnagi.2025.1579208 40799366 PMC12339460

[B29] Gao F. Zhang Y. Hou X. Tao Z. Ren H. Wang G. (2020). Dependence of PINK1 accumulation on mitochondrial redox system. Aging Cell 19, e13211. doi: 10.1111/acel.13211 32779864 PMC7511888

[B32] García-Santamarina S. Boronat S. Hidalgo E. (2014). Reversible cysteine oxidation in hydrogen peroxide sensing and signal transduction. Biochemistry 53, 2560–2580. doi: 10.1021/bi401700f 24738931

[B33] Gautier C. A. Kitada T. Shen J. (2008). Loss of PINK1 causes mitochondrial functional defects and increased sensitivity to oxidative stress. Proc. Natl. Acad. Sci. U.S.A. 105, 11364–11369. doi: 10.1073/pnas.0802076105 18687901 PMC2516271

[B34] Goiran T. Duplan E. Rouland L. El Manaa W. Lauritzen I. Dunys J. . (2018). Nuclear p53-mediated repression of autophagy involves PINK1 transcriptional down-regulation. Cell Death Differ. 25, 873–884. doi: 10.1038/s41418-017-0016-0 29352272 PMC5943347

[B35] Guardia-Laguarta C. Liu Y. Lauritzen K. H. Erdjument-Bromage H. Martin B. Swayne T. C. . (2019). Pink1 content in mitochondria is regulated by ER-associated degradation. J. Neurosci. 39, 7074–7085. doi: 10.1523/JNEUROSCI.2951-18.2019 31300519 PMC6733537

[B36] Gupta P. Sharma G. Lahiri A. Barthwal M. K. (2022). Foxo3a acetylation regulates PINK1, mitophagy, inflammasome activation in murine palmitate-conditioned and diabetic macrophages. J. Leukoc. Biol. 111, 611–627. doi: 10.1002/JLB.3A0121-049RR 34288093

[B38] Heo A. J. Ji C. H. Kwon Y. T. (2023). The Cys/N-degron pathway in the ubiquitin-proteasome system and autophagy. Trends Cell Biol. 33, 247–259. doi: 10.1016/j.tcb.2022.07.004 35945077

[B39] Holloszy J. O. Narahara H. T. (1965). Oxidation of pyruvate by isolated frog muscle mitochondria stimulated by exercise. Science 150, 1122–1123. doi: 10.1126/science.150.3699.1122 5852969

[B40] Imberechts D. Kinnart I. Wauters F. Terbeek J. Manders L. Wierda K. . (2022). Dj-1 is an essential downstream mediator in PINK1/parkin-dependent mitophagy. Brain 145, 4368–4384. doi: 10.1093/brain/awac274 36039535 PMC9762950

[B42] Ji L. L. Yeo D. Kang C. Zhang T. (2020). The role of mitochondria in redox signaling of muscle homeostasis. J. Sport Health Sci. 9, 386–393. doi: 10.1016/j.jshs.2019.10.001 32780692 PMC7498629

[B43] Jia H. Song Y. Hua Y. Li K. Li S. Wang Y. (2025). Molecular mechanism of aerobic exercise ameliorating myocardial mitochondrial injury in mice with heart failure. Int. J. Mol. Sci. 26, 2136. doi: 10.3390/ijms26052136 40076760 PMC11901053

[B41] Jin S. M. Lazarou M. Wang C. Kane L. A. Narendra D. P. Youle R. J. (2010). Mitochondrial membrane potential regulates PINK1 import and proteolytic destabilization by PARL. J. Cell Biol. 191, 933–942. doi: 10.1083/jcb.201008084 21115803 PMC2995166

[B44] Kang J. Liu M. Yang Q. Dang X. Li Q. Wang T. . (2025). Exercise training exerts beneficial effects on Alzheimer's disease through multiple signaling pathways. Front. Aging Neurosci. 17, 1558078. doi: 10.3389/fnagi.2025.1558078 40469843 PMC12133837

[B45] Khoramipour K. Soltany A. Khosravi P. Rezaei M. H. Madadizadeh E. García-Chico C. . (2024). High intensity interval training as a therapy: mitophagy restoration in breast cancer. Arch. Biochem. Biophys. 762, 110213. doi: 10.1016/j.abb.2024.110213 39515549

[B46] Kolodziej F. O'Halloran K. D. (2021). Re-evaluating the oxidative phenotype: can endurance exercise save the Western world? Antioxidants (Basel) 10, 609. doi: 10.3390/antiox10040609 33921022 PMC8071436

[B47] Lai L. Sun J. Tarafdar S. Liu C. Murphy E. Kim G. . (2019). Loss of methionine sulfoxide reductases increases resistance to oxidative stress. Free Radic. Biol. Med. 145, 374–384. doi: 10.1016/j.freeradbiomed.2019.10.005 31606431 PMC6891793

[B49] Laker R. C. Drake J. C. Wilson R. J. Lira V. A. Lewellen B. M. Ryall K. A. . (2017). Ampk phosphorylation of Ulk1 is required for targeting of mitochondria to lysosomes in exercise-induced mitophagy. Nat. Commun. 8, 548. doi: 10.1038/s41467-017-00520-9 28916822 PMC5601463

[B50] Lazarou M. Jin S. M. Kane L. A. Youle R. J. (2012). Role of PINK1 binding to the TOM complex and alternate intracellular membranes in recruitment and activation of the E3 ligase Parkin. Dev. Cell 22, 320–333. doi: 10.1016/j.devcel.2011.12.014 22280891 PMC3288275

[B48] Lee K. H. Cha M. Lee B. H. (2020). Neuroprotective effect of antioxidants in the brain. Int. J. Mol. Sci. 21, 7152. doi: 10.3390/ijms21197152 32998277 PMC7582347

[B55] Li H. R. Liu Q. Zhu C. L. Sun X. Y. Sun C. Y. Yu C. M. . (2023). β-Nicotinamide mononucleotide activates NAD+/SIRT1 pathway and attenuates inflammatory and oxidative responses in the hippocampus regions of septic mice. Redox Biol. 63, 102745. doi: 10.1016/j.redox.2023.102745 37201414 PMC10206198

[B56] Li H. Qin S. Liang Q. Xi Y. Bo W. Cai M. . (2021). Exercise training enhances myocardial mitophagy and improves cardiac function via irisin/FNDC5-PINK1/Parkin pathway in MI mice. Biomedicines. 9, 701. doi: 10.3390/biomedicines9060701 34205641 PMC8234442

[B51] Lin Z. Chen C. Yang D. Ding J. Wang G. Ren H. (2021). DJ-1 inhibits microglial activation and protects dopaminergic neurons *in vitro* and *in vivo* through interacting with microglial p65. Cell Death Dis. 12, 715. doi: 10.1038/s41419-021-04000-8 34274951 PMC8286256

[B54] Lin Y. Wen D. T. (2026). Pink1 at the crossroads of aging, exercise, and diet in Parkinson's disease: a mechanistic review. Front. Aging Neurosci. 18, 1738559. doi: 10.3389/fnagi.2026.1738559 42038693 PMC13106505

[B53] Liu H. W. Chang S. J. (2018). Moderate exercise suppresses NF-κB signaling and activates the SIRT1-AMPK-PGC1α axis to attenuate muscle loss in diabetic db/db mice. Front. Physiol. 9, 636. doi: 10.3389/fphys.2018.00636 29896118 PMC5987703

[B52] Liu C. He W. Zhang J. (2025). Exercise regulates mitophagy to alleviate parkinsonian neurodegeneration. Front. Aging Neurosci. 17, 1678460. doi: 10.3389/fnagi.2024.1678460 41356234 PMC12679300

[B57] Luk H. Y. Jiwan N. C. Appell C. R. Levitt D. E. Vingren J. L. (2022). Sex-specific mitochondrial dynamics and mitophagy response to muscle damage. Physiol. Rep. 10, e15230. doi: 10.14814/phy2.15230 35611770 PMC9131605

[B58] Marchenko N. D. Moll U. M. (2014). Mitochondrial death functions of p53. Mol. Cell. Oncol. 1, e955995. doi: 10.4161/23723548.2014.955995 27308326 PMC4905191

[B59] Markaki M. Tsagkari D. Tavernarakis N. (2023). Mitophagy and long-term neuronal homeostasis. J. Cell Sci. 136, jcs260638. doi: 10.1242/jcs.260638 37278219

[B60] Marques-Aleixo I. Santos-Alves E. Balça M. M. Rizo-Roca D. Moreira P. I. Oliveira P. J. . (2015). Physical exercise improves brain cortex and cerebellum mitochondrial bioenergetics and alters apoptotic, dynamic and auto(mito)phagy markers. Neuroscience 301, 480–495. doi: 10.1016/j.neuroscience.2015.06.027 26116519

[B61] McCoin C. S. Franczak E. Deng F. Pei D. Ding W. X. Thyfault J. P. (2022). Acute exercise rapidly activates hepatic mitophagic flux. J. Appl. Physiol. (1985) 132, 862–873. doi: 10.1152/japplphysiol.00690.2021 35142562 PMC8934677

[B62] Mei Y. Zhang Y. Yamamoto K. Xie W. Mak T. W. You H. (2009). FOXO3a-dependent regulation of Pink1 (Park6) mediates survival signaling in response to cytokine deprivation. Proc. Natl. Acad. Sci. U.S.A. 106, 5153–5158. doi: 10.1073/pnas.0813337106 19276113 PMC2654023

[B64] Miyama A. Saito Y. Yamanaka K. Hayashi K. Hamakubo T. Noguchi N. (2011). Oxidation of DJ-1 induced by 6-hydroxydopamine decreasing intracellular glutathione. PloS One 6, e27883. doi: 10.1371/journal.pone.0027883 22132160 PMC3221727

[B65] Moghazy H. M. Seham A. A. Mahmoud M. Gebril S. M. Monir D. M. (2026). Involvement of the PINK1/PARKIN pathway in enhancing mitochondrial function and mitophagy in reserpine-induced fibromyalgia mice through strength exercise and coenzyme Q10. Eur. J. Appl. Physiol. 126, 2219–2234. doi: 10.1007/s00421-025-05990-0 41329351 PMC13171688

[B66] Murata H. Takamatsu H. Liu S. Kataoka K. Huh N. H. Sakaguchi M. (2015). NRF2 regulates PINK1 expression under oxidative stress conditions. PloS One 10, e0142438. doi: 10.1371/journal.pone.0142438 26555609 PMC4640816

[B63] Murphy K. T. Lynch G. S. (2023). Impaired skeletal muscle health in Parkinsonian syndromes: clinical implications, mechanisms and potential treatments. J. Cachexia Sarcopenia Muscle. 14, 1987–2002. doi: 10.1002/jcsm.13312 37574254 PMC10570091

[B67] Nambu H. Takada S. Maekawa S. Matsumoto J. Kakutani N. Furihata T. . (2021). Inhibition of xanthine oxidase in the acute phase of myocardial infarction prevents skeletal muscle abnormalities and exercise intolerance. Cardiovasc. Res. 117, 805–819. doi: 10.1093/cvr/cvaa071 32402072

[B68] Navarro A. Gomez C. López-Cepero J. M. Boveris A. (2004). Beneficial effects of moderate exercise on mice aging: survival, behavior, oxidative stress, and mitochondrial electron transfer. Am. J. Physiol. Regul. Integr. Comp. Physiol. 286, R505–R511. doi: 10.1152/ajpregu.00208.2003 14615275

[B69] Nikolaidis M. G. Kyparos A. Spanou C. Paschalis V. Theodorou A. A. Vrabas I. S. (2012). Redox biology of exercise: an integrative and comparative consideration of some overlooked issues. J. Exp. Biol. 215, 1615–1625. doi: 10.1242/jeb.067470 22539728

[B70] Okatsu K. Sato Y. Yamano K. Matsuda N. Negishi L. Takahashi A. . (2018). Structural insights into ubiquitin phosphorylation by PINK1. Sci. Rep. 8, 10382. doi: 10.1038/s41598-018-28656-8 29991771 PMC6039469

[B72] Pickrell A. M. Youle R. J. (2015). The roles of PINK1, parkin, and mitochondrial fidelity in Parkinson's disease. Neuron 85, 257–273. doi: 10.1016/j.neuron.2014.12.007 25611507 PMC4764997

[B73] Pilegaard H. Saltin B. Neufer P. D. (2003). Exercise induces transient transcriptional activation of the PGC-1alpha gene in human skeletal muscle. J. Physiol. 546, 851–858. doi: 10.1113/jphysiol.2002.034850 12563009 PMC2342594

[B74] Poljsak B. Milisav I. (2016). NAD+ as the link between oxidative stress, inflammation, caloric restriction, exercise, DNA repair, longevity, and health span. Rejuvenation Res. 19, 406–415. doi: 10.1089/rej.2015.1767 26725653

[B75] Powers S. K. Jackson M. J. (2008). Exercise-induced oxidative stress: cellular mechanisms and impact on muscle force production. Physiol. Rev. 88, 1243–1276. doi: 10.1152/physrev.00031.2007 18923182 PMC2909187

[B71] Powers S. K. Radak Z. Ji L. L. Jackson M. (2024). Reactive oxygen species promote endurance exercise-induced adaptations in skeletal muscles. J. Sport Health Sci. 13, 780–792. doi: 10.1016/j.jshs.2024.05.001 38719184 PMC11336304

[B77] Quinlan C. L. Gerencser A. A. Treberg J. R. Brand M. D. (2011). The mechanism of superoxide production by the antimycin-inhibited mitochondrial Q-cycle. J. Biol. Chem. 286, 31361–31372. doi: 10.1074/jbc.M111.267898 21708945 PMC3173136

[B76] Quinn P. M. J. Moreira P. I. Ambrósio A. F. Alves C. H. (2020). PINK1/PARKIN signalling in neurodegeneration and neuroinflammation. Acta Neuropathol. Commun. 8, 189. doi: 10.1186/s40478-020-01062-w 33168089 PMC7654589

[B78] Rahman F. A. Graham M. Q. Adam A. M. Juracic E. S. Tupling A. R. Quadrilatero J. (2025). Mitophagy is required to protect against excessive skeletal muscle atrophy following hindlimb immobilization. J. Biomed. Sci. 32, 29. doi: 10.1186/s12929-025-01118-w 39979946 PMC11844018

[B79] Rosencrans W. M. Lee R. W. McGraw L. Horsburgh I. Wang T. Y. Quan B. . (2025). Putative PINK1/Parkin activators lower the threshold for mitophagy by sensitizing cells to mitochondrial stress. Sci. Adv. 11, eady0240. doi: 10.1126/sciadv.ady0240 40864725 PMC12383277

[B88] Sasaki Y. Ikeda Y. Uchikado Y. Akasaki Y. Sadoshima J. Ohishi M. (2021). Estrogen plays a crucial role in Rab9-dependent mitochondrial autophagy, delaying arterial senescence. J. Am. Heart Assoc. 10, e019310. doi: 10.1161/jaha.120.019310 33719502 PMC8174372

[B80] Seabright A. P. Lai Y. C. (2020). Regulatory roles of PINK1-Parkin and AMPK in ubiquitin-dependent skeletal muscle mitophagy. Front. Physiol. 11, 608474. doi: 10.3389/fphys.2020.608474 33343399 PMC7744660

[B89] Sebori R. Kuno A. Hosoda R. Hayashi T. Horio Y. (2018). Resveratrol decreases oxidative stress by restoring mitophagy and improves the pathophysiology of dystrophin-deficient mdx mice. Oxid. Med. Cell. Longev. 2018, 9179270. doi: 10.1155/2018/9179270 30510631 PMC6231358

[B81] Sekine S. Kanamaru Y. Koike M. Nishihara A. Okada M. Kinoshita H. . (2012). Rhomboid protease PARL mediates the mitochondrial membrane potential loss-induced cleavage of PGAM5. J. Biol. Chem. 287, 34635–34645. doi: 10.1074/jbc.M112.370437 22915595 PMC3464569

[B83] Sekine S. Youle R. J. (2018). PINK1 import regulation; a fine system to convey mitochondrial stress to the cytosol. BMC Biol. 16, 2. doi: 10.1186/s12915-017-0470-7 29325568 PMC5795276

[B84] Shao S. Lu H. Zu R. Chen Y. Peng Z. Peng Q. . (2026). Pharmacological regulation of mitophagy by natural plant products as a therapeutic target for Alzheimer's disease. Phytother. Res. 40, 248–266. doi: 10.1002/ptr.8105 41272400

[B86] Shi S. Y. Lu S. Y. Sivasubramaniyam T. Revelo X. S. Cai E. P. Luk C. T. . (2015). DJ-1 links muscle ROS production with metabolic reprogramming and systemic energy homeostasis in mice. Nat. Commun. 6, 7415. doi: 10.1038/ncomms8415 26077864 PMC4490365

[B85] Shi G. McQuibban G. A. (2017). The mitochondrial rhomboid protease PARL is regulated by PDK2 to integrate mitochondrial quality control and metabolism. Cell Rep. 18, 1458–1472. doi: 10.1016/j.celrep.2017.01.029 28178523

[B87] Singh F. Wilhelm L. Prescott A. R. Ostacolo K. Zhao J. F. Ogmundsdottir M. H. . (2024). PINK1 regulated mitophagy is evident in skeletal muscles. Autophagy Rep. 3, 2326402. doi: 10.1080/27694127.2024.2326402 38988500 PMC7616148

[B90] Smith A. J. Phipps W. R. Thomas W. Schmitz K. H. Kurzer M. S. (2013). The effects of aerobic exercise on estrogen metabolism in healthy premenopausal women. Cancer Epidemiol. Biomarkers Prev. 22, 756–764. doi: 10.1158/1055-9965.epi-12-1325 23652373 PMC3648856

[B91] Steiner J. L. Murphy E. A. McClellan J. L. Carmichael M. D. Davis J. M. (2011). Exercise training increases mitochondrial biogenesis in the brain. J. Appl. Physiol. (1985) 111, 1066–1071. doi: 10.1152/japplphysiol.00343.2011 21817111

[B82] Sun L. Wu L. Xu Z. Zeng W. Wang Y. (2025). Running exercise alleviates depressive-like behaviors through the activation of PINK1-Parkin mediated mitophagy in mice exposed to chronic social defeat stress. Psychiatry Res. 352, 116714. doi: 10.1016/j.psychres.2025.116714 40912062

[B92] Takahashi M. Hood D. A. (2012). Plasticity of TOM complex assembly in skeletal muscle mitochondria in response to chronic contractile activity. FASEB J. 26, 1258–1267. doi: 10.1096/fj.11-197475 22142511

[B94] Tang S. Geng Y. Lin Q. (2024). The role of mitophagy in metabolic diseases and its exercise intervention. Front. Physiol. 15, 1339128. doi: 10.3389/fphys.2024.1339128 38348222 PMC10859464

[B93] Tang P. Zeng Q. Li Y. Wang J. She M. (2025). The mitochondrial LONP1 protease: molecular targets and role in pathophysiology. Mol. Biol. Rep. 52, 401. doi: 10.1007/s11033-025-10492-3 40249453

[B95] Thirupathi A. de Souza C. T. (2017). Multi-regulatory network of ROS: the interconnection of ROS, PGC-1 alpha, and AMPK-SIRT1 during exercise. J. Physiol. Biochem. 73, 487–494. doi: 10.1007/s13105-017-0576-y 28707280

[B96] Unoki M. Nakamura Y. (2001). Growth-suppressive effects of BPOZ and EGR2, two genes involved in the PTEN signaling pathway. Oncogene 20, 4457–4465. doi: 10.1038/sj.onc.1204608 11494141

[B97] Valente E. M. Abou-Sleiman P. M. Caputo V. Muqit M. M. Harvey K. Gispert S. . (2004). Hereditary early-onset Parkinson's disease caused by mutations in PINK1. Science 304, 1158–1160. doi: 10.1126/science.1096284 15087508

[B98] Van Laar V. S. Berman S. B. (2013). The interplay of neuronal mitochondrial dynamics and bioenergetics: implications for Parkinson's disease. Neurobiol. Dis. 51, 43–55. doi: 10.1016/j.nbd.2012.05.015 22668779 PMC4015731

[B99] Varshavsky A. (2019). N-degron and C-degron pathways of protein degradation. Proc. Natl. Acad. Sci. U.S.A. 116, 358–366. doi: 10.1073/pnas.1816596116 30622213 PMC6329975

[B100] Wadley A. J. Aldred S. Coles S. J. (2016). An unexplored role for Peroxiredoxin in exercise-induced redox signalling? Redox Biol. 8, 51–58. doi: 10.1016/j.redox.2015.12.003 26748042 PMC4712319

[B101] Xu Z. Fu T. Guo Q. Zhou D. Sun W. Zhou Z. . (2022). Disuse-associated loss of the protease LONP1 in muscle impairs mitochondrial function and causes reduced skeletal muscle mass and strength. Nat. Commun. 13, 894. doi: 10.1038/s41467-022-28556-9 35173176 PMC8850466

[B102] Yamano K. Youle R. J. (2013). PINK1 is degraded through the N-end rule pathway. Autophagy 9, 1758–1769. doi: 10.4161/auto.24633 24121706 PMC4028335

[B103] Yang J. L. Lin Y. T. Chuang P. C. Bohr V. A. Mattson M. P. (2014). BDNF and exercise enhance neuronal DNA repair by stimulating CREB-mediated production of apurinic/apyrimidinic endonuclease 1. Neuromolecular Med. 16, 161–174. doi: 10.1007/s12017-013-8274-1 24114393 PMC3948322

[B104] Yu M. Jiang X. Zhang Y. Zhang W. Wang T. Wang J. . (2025). Mitophagy as a therapeutic target for exercise-induced fatigue: modulation by natural compounds and mechanistic insights. Front. Physiol. 16, 1664909. doi: 10.3389/fphys.2025.1664909 41245270 PMC12615170

[B105] Zanini G. Selleri V. Malerba M. Solodka K. Sinigaglia G. Nasi M. . (2023). The role of Lonp1 on mitochondrial functions during cardiovascular and muscular diseases. Antioxidants (Basel) 12, 598. doi: 10.3390/antiox12030598 36978846 PMC10045650

[B106] Zhao N. Zhang X. Li B. Wang J. Zhang C. Xu B. (2023). Treadmill exercise improves PINK1/Parkin-mediated mitophagy activity against Alzheimer's disease pathologies by upregulated SIRT1-FOXO1/3 axis in APP/PS1 mice. Mol. Neurobiol. 60, 277–291. doi: 10.1007/s12035-022-03074-7 36261693

[B107] Zorova L. D. Popkov V. A. Plotnikov E. Y. Silachev D. N. Pevzner I. B. Jankauskas S. S. . (2018). Mitochondrial membrane potential. Anal. Biochem. 552, 50–59. doi: 10.1016/j.ab.2017.07.009 28711444 PMC5792320

